# Combination of SB431542, Chir9901, and Bpv as a novel supplement in the culture of umbilical cord blood hematopoietic stem cells

**DOI:** 10.1186/s13287-020-01945-8

**Published:** 2020-11-09

**Authors:** Morteza Zarrabi, Elaheh Afzal, Mohammad Hassan Asghari, Marzieh Ebrahimi

**Affiliations:** 1 Department of Stem Cells and Developmental Biology, Cell Science Research Center, Royan Institute for Stem Cell Biology and Technology, ACECR, P.O. Box, Tehran, 19395-4644 Iran; 2 Royan Stem Cell Technology Company, Cord Blood Bank, Tehran, Iran; 3 Animal Core Facility, Reproductive Biomedicine Research Center, Royan Institute for Animal Biotechnology, ACECR, Tehran, Iran

**Keywords:** Cord blood, Hematopoietic stem cells, Small molecules, Ex vivo expansion

## Abstract

**Background:**

Small molecule compounds have been well recognized for their promising power in the generation, expansion, and maintenance of embryonic or adult stem cells. The aim of this study was to identify a novel combination of small molecules in order to optimize the ex vivo expansion of umbilical cord blood-derived CD34^+^ cells.

**Methods:**

Considering the most important signaling pathways involved in the self-renewal of hematopoietic stem cells, CB-CD34^+^ cells were expanded with cytokines in the presence of seven small molecules including SB, PD, Chir, Bpv, Pur, Pμ, and NAM. The eliminativism approach was used to find the best combination of selected small molecules for effective ex vivo expansion of CD34^+^ cell. In each step, proliferation, self-renewal, and clonogenic potential of the expanded cells as well as expression of some hematopoietic stem cell-related genes were studied. Finally, the engraftment potential of expanded cells was also examined by the mouse intra-uterine transplantation model.

**Results:**

Our data shows that the simultaneous use of SB431542 (TGF-β inhibitor), Chir9901 (GSK3 inhibitor), and Bpv (PTEN inhibitor) resulted in a 50-fold increase in the number of CD34^+^CD38^−^ cells. This was further reflected in approximately 3 times the increase in the clonogenic potential of the small molecule cocktail-expanded cells. These cells, also, showed a 1.5-fold higher engraftment potential in the peripheral blood of the NMRI model of in utero transplantation. These results are in total conformity with the upregulation of HOXB4, GATA2, and CD34 marker gene as well as the CXCR4 homing gene.

**Conclusion:**

Taken together, our findings introduce a novel combination of small molecules to improve the yield of existing protocols used in the expansion of hematopoietic stem cells.

## Introduction

The umbilical cord blood (UCB) as one of the most valuable and convenient sources of hematopoietic stem cells (HSCs) has a great potential for the treatment of various hematological, non-hematological disorders, and cancers [1–4]. However, the limited number of HSCs in a UCB unit has limited its use to the young patients. In this regard, ex vivo expansion is one of the main solutions proposed to acquire a sufficient number of HSCs [5, 6]. Therefore, in recent years, many efforts have been made to identify the factors affecting the self-renewal of the umbilical cord CD34^+^ cells as well as the more primitive hematopoietic stem and progenitors, CD34^+^CD38^−^ cells [7].

The use of small molecules in the field of hematopoietic stem cell research has grown rapidly in recent years, as they are good tools for controlling the variety of cellular processes [8]. There are different approaches to select small molecules in HSC expansion; induction of self-renewal [9], inhibition of lineage commitment differentiation [10], and inhibition of HSC apoptosis [11, 12]. In the present study, we hypothesized that the best expansion is achieved when the proliferation, survival, and self-renewal pathways are induced, while the apoptosis and differentiation pathways are inhibited, simultaneously. Therefore, through data mining, a limited set of seven small molecules were selected which are as follows:

- SB431542 (SB) and Purmorphamin that respectively regulate TGFβ and SHh pathways and are associated with the proliferation of HSCs [13, 14].
- PD0325901 (PD) and Chir9901 (Chir) that regulate Wnt/β-catenin and ERK pathways and play important role in HSCs differentiation [15–18].
- Bisperoxovanadium (Bpv) and Pifithrin-μ (Pμ) that are associated with the pathways related to HSC survival like Akt and P53 [19, 20].
- Nicotinamide that facilitates the transcriptional epigenetic changes of chromatin [21].

The main question was whether a cocktail of these small molecules along with SCF, TPO, and Flt3L, the common cytokines which are basically used in the culture media of hematopoietic stem cells [22], could improve the self-renewal and transplantation potential of ex vivo expanded cells. To find the best combination, the eliminative approach was used, in which the components of a system are removed one by one; then, the interaction between the other components is investigated and the system is re-constructed. Here, we report that a cocktail consisting of SB, Chir, and Bpv is effective in promoting the cord blood hematopoietic stem cell proliferation while their stemness and in vivo engraftment potential maintained.

## Methods

### Ethical approval

All the experiments in this study were reviewed and approved by the Research Ethics Committee of the Royan Institute and were conducted in accordance with the ethical principles and the national norms and standards for conducting the Medical Research in Iran (IR.ACECR.ROYAN.REC.1398.189).

### Isolation of CD34^+^ cells

A schematic illustration of the procedure was shown in Supplementary Fig. 1. Umbilical cord blood (UCB) samples were obtained from the Royan Cord Blood Bank. The collection of UCB was performed with the informed consent of the mother. Mononuclear cells were isolated using hydroxyethyl starch (Grifols, Spain) followed by LymphoprepTM (Stem cell Technology Inc.) density-gradient centrifugation. To isolate CD34^+^ cell, immuno-magnetic selection kit (Miltenyi Biotec, Germany) was used. Highly purified (> 90%) CD34^+^ cells were confirmed by flow cytometry (Partec PAS system, USA) and then prepared to expand in different culture condition.

### MTS assay

To determine the maximum tolerated dose of small molecules, the MTS assay was performed. At first, the initial concentration of small molecules was selected based on the previous studies (Supplementary Table 1). Two-point lower and two-higher concentrations were selected for cytotoxic assay. Briefly, cells were seeded into 96-well plates at a density of 1.0 × 10^4^ cells/well in different concentrations of small molecules for 48 h. Control cells received an equal amount of 10% FBS-IMDM medium without any small molecule. Then, 100 μL of MTS (promega) was subsequently added to each well and then incubated in the dark at 37 °C for at least 1 h. The absorbance was measured at 490 nm. All groups were normalized to the same control group, and significant data was calculated using one-way ANOVA. All data were collected from five independent experiments.

### Ex vivo expansion

Umbilical cord blood CD34^+^ cells were cultured for 10 days in the serum-free StemSpan™ medium (Stem Cell Technology Inc.) supplemented with 100 ng/mL stem cell factor (SCF), 100 ng/mL Fms-related tyrosine kinase 3 ligand (Flt3-L), and 50 ng/mL thrombopoietin (TPO), all from R&D. Seven small molecules: SB (10 μM), Bpv (5 μM), NAM (2.5 μM), Pur (4 μM)PD (0.25 μM), Chir (0.37 μM), and Pμ (2.5 μM) were added to the media. CD34^+^ cells treated just with cytokines served as a positive control. The cells were maintained at 37 °C in a humidified atmosphere containing 5% of CO_2_ and passaged every 3 days. Total nuclear cells were enumerated by trypan blue, and cellular expansion fold was calculated based on the initial inputs.

### Immunophenotyping of expanded cells

Cells were collected and stained with an anti-human CD34 monoclonal antibody conjugated to phycoerythrin (PE; BD Pharmingen™) and an anti-human CD38 monoclonal antibody conjugated to allophycocyanin (PerCP-Cy™5.5, BD Pharmingen™), together or separately. The appropriate isotype control antibodies were used for setting the Partec PAS system. At least 10^4^ events were acquired and data was analyzed using FlowMax software.

### Colony-forming assay

Colony-forming units (CFUs) were generated by seeding 300 expanded cells into 1.1 ml methylcellulose media (H4434, Stem Cell Technologies, Canada) diluted with IMDM + 2% FBS at a ratio of 1/10. The colonies including burst-forming unit-erythroid (BFU-E), CFU granulocyte-macrophage (CFU-GM), and CFU granulocyte-erythrocyte-macrophage-megakaryocyte (CFUs-GEMM) were scored based on their morphology on day 14–16 at ×4 magnification under an inverted microscope. All experiments were done as duplicates; all colonies were counted by an expertise in hematological colony counts and a mean of at least three independent experiments was reported.

### RNA extraction and qPCR

Total RNA was isolated using QIAzol lysis reagent. The integrity and quality of RNA samples were checked using a Nano Drop (ND-1000) spectrophotometer. One microgram of the total RNA was subjected to reverse transcription using oligo-dT and PrimeScriptTM 1st-strand cDNA kit (Takara, Japan). Transcript levels were determined using the SYBR Green master mix and Corbett Rotor-Gene 6000. The GAPDH-normalized transcript data are shown as relative expression levels in the small molecules cocktail compared to the corresponding level in a positive control group. The primer sequences for qRT-PCR are listed in Supplementary Table 2.

### Animals and xeno-transplantation study

Xeno-transplantation was done as reported previously by our group [23]. Briefly, on embryonic days E11.5–E13.5, each NMRI embryo injected intraperitoneally with 2–3 × 10^4^ fresh CD34^+^ cells or their entire progeny following 10 days of expansion. To repopulate CD34^+^ cells, newborn mice were treated with human hematopoietic growth factors (interleukin 3 (IL-3) 4 ng/g, SCF (4 ng/g), and granulocyte colony-stimulating factor (G-CSF) 50 ng/g), beginning at 3 weeks of age. Then, the percentage of human CD45 cells (as a marker of human chimerism) in the peripheral blood of recipients was assessed monthly up to 4 months post birth. After staining the peripheral blood with anti-human CD45, at least 10^5^ cells were analyzed on a Partec system. Engraftment is defined as the detection of 0.2% or more human CD45 cells.

### Statistical analysis

All the data were presented as mean ± SD of at least three different biological replicates. One way ANOVA was used to analyze the MTS assay data and the two-tailed Student’s *t* test was used for statistical comparisons between the groups. *P* < 0.05 was considered a statistically significant difference.

## Results

### Optimization of small molecule doses for HSC expansion

**Fig. 1:**
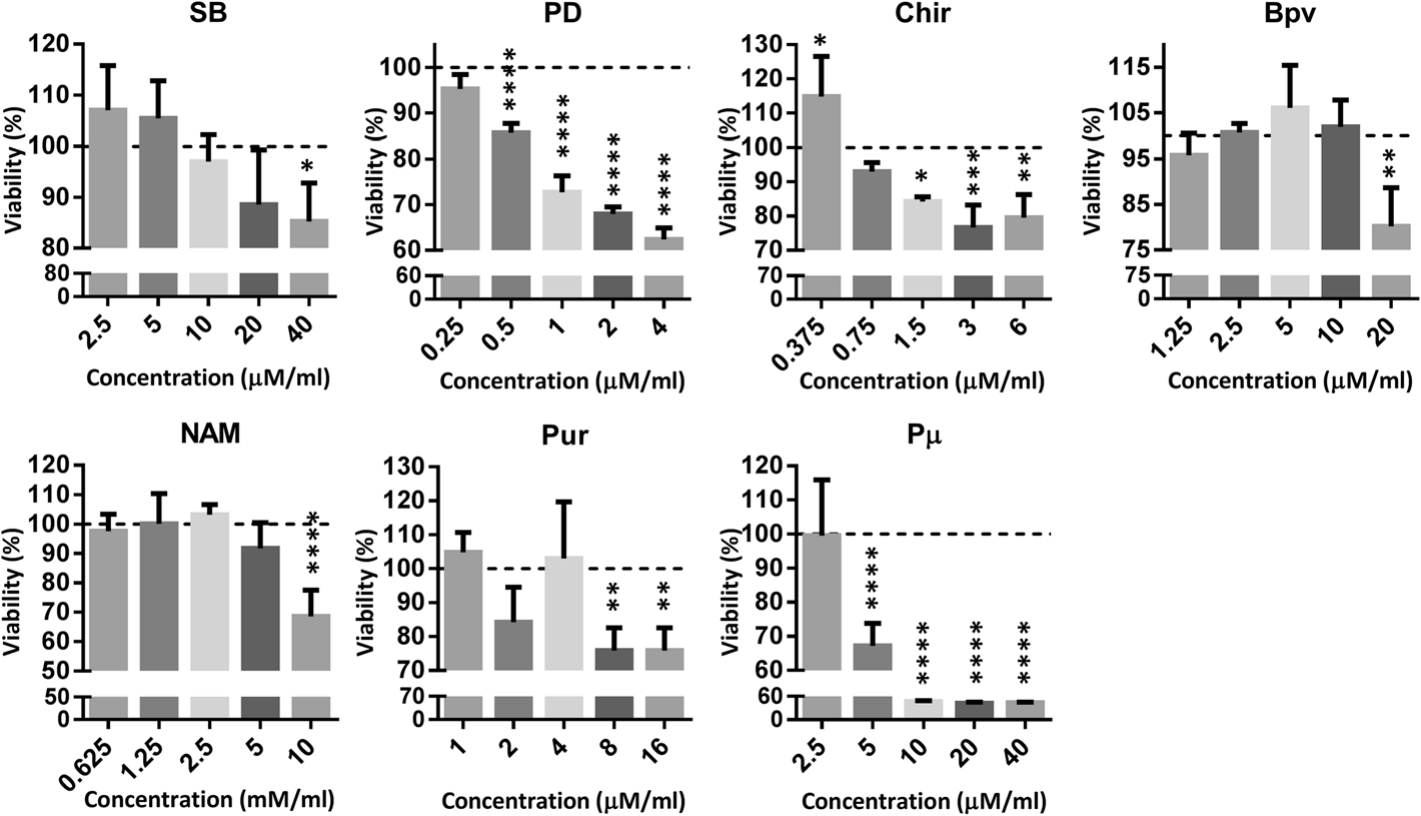
Dose finding and optimization for small molecules (SB, Bpv, NAM, Pur, PD, Chir, Pμ). In each graph, the middle column corresponds to the reference concentration of the small molecules based on the literatures. Cell viability was measured by MTS assay post 48 h incubation with small molecules. The negative control in each group was used for the normalization of data. Bars indicated as mean ± SD at least five independent replicates. *P ≤ 0.05, **P ≤ 0.01, ***P ≤ 0.001, ****P ≤ 0.0001

The proper concentration of selected small molecules which was not cytotoxic for CD34^+^ cells was determined using MTS assay (Fig. 1). In consistent with the other studies, CD34^+^ cells cultivated in SB (10 μM), Bpv (5 μM), NAM (2.5 μM), and Pur (4 μM) were viable. However, predetermined concentrations of PD (1 μM), Chir (3 μM), and Pμ (10 μM) were toxic for UCB-HSCs. Therefore, lower concentrations of PD (0.25 μM), Chir (0.37 μM), and Pμ (2.5 μM) were added to the culture medium.

### SB, Chir, and Bpv are sufficient for ex vivo expansion of UCB-CD34^+^ cells

**Fig. 2:**
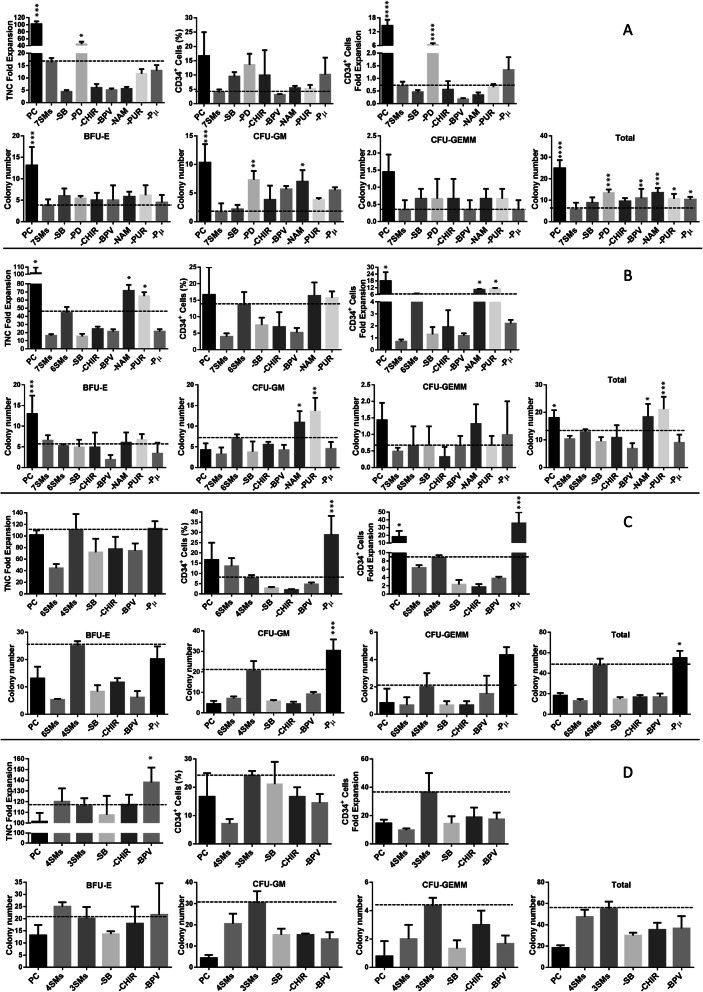
Characterization of expanded UCB-CD34^+^ cells in the presence/absence of different combinations of small molecules. TNC fold expansion, CD34^+^ cells percentage, fold expansion of CD34^+^ cells, and colony-forming potential of UCB-CD34^+^ cells was evaluated in each experiment. **a** 7 SM cocktail (SB, PD, Chir, Bpv, NAM, Pur, Pμ) and its derivative groups **b** 6 SMs cocktail (SB, Chir, Bpv, NAM, Pur, Pμ) and its derivative groups **c** 4 SMs cocktail (SB, Chir, Bpv, Pμ) and its derivative groups **d** 3 SMs cocktail (SB, Chir, Bpv). CD34^+^ cells cultivated in presence of SCF, FLT3L, and TPO were used as a positive control. Fold expansion was determined by dividing the total number of viable cells expressing the phenotype at the end of the culture by the input number of viable cells expressing the same phenotype (*n* = 3). A statistically significant difference compared with a positive control group, *P ≤ 0.05, **P ≤ 0.01,***P ≤ 0.001

We next did some serial experiments (Supplementary Fig. 1). In the first round of experiments, isolated UCB-CD34^+^ cells were cultured in the presence of cytokines (SCF, TPO, and Flt3L) and selected small molecules. In the other groups, small molecules were deleted one by one from the pool of 7 SMs. Although, individual removal of SB, Chir, Bpv, Pur, NAM, and Pμ did not make significant differences in total nuclear cell (TNC) number compared to the 7SM group, removal of PD yielded an increased total number of mononuclear cells (Fig. 2a). The precise effect of PD on ex vivo expansion of CD34^+^ cells has been discussed before [16]. An additional round of small molecules removal showed that the deletion of NAM and Pur from the cocktail increased the fold expansion of TNCs and CD34^+^ cells. Furthermore, the groups lacking NAM and Pur had a higher colony-forming potential, especially CFU-GM, compared to other groups containing small molecules (Fig. 2b). In the next round, by removing Pμ, the number of CD34^+^ cells, CFU-GM, and CFU-GEMM colonies was increased significantly compared to the PC group (Fig. 2c). In the final round, removal of SB, Chir, or Bpv reduced the expansion of CD34^+^CD38^−^ cells and abolished the formation of CFU-GM and CFU-GEMM colonies, showing that these are essential for CD34^+^ cell expansion (Fig. 2d). Although, there was no significant difference between the 3SMCs and the positive control in terms of TNC expansion, removal of Bpv slightly increased the TNC fold expansion compared to the 3SM group (118 to 140). Moreover, the exclusion of each of the remaining three SMs (SB, Chir, or Bpv) had a dramatic negative impact on the expansion CD34^+^CD38^−^ cells. Expansion with these three SMs (SB, Chir, and Bpv) produced a 2.7-fold increase in the number of CD34^+^CD38^−^ cells relative to a positive control (17 vs. 47). Finally, a CFU assay was performed to determine if the optimal SM cocktail actually promotes the expansion of hUCB-HPCs. As shown in Fig. 2d, the number of total CFUs increased more than 3-fold when CD34^+^ cells was expanded in the presence of SB, Chir, and Bpv for 10 days compared to the positive control. The expanded cells generated significantly more BFU and CFU-GM than the positive control (*p* < 0.01). However, the number of GEMMs in the SM group was slightly greater than that of the PC, but the difference was not statistically significant (*p* > 0.05).

### The ability of 3SM cocktail to enhance the short-term engraftment potential of ex vivo expanded CD34^+^ cells in the in utero transplanted NMRI mice

**Fig. 3:**
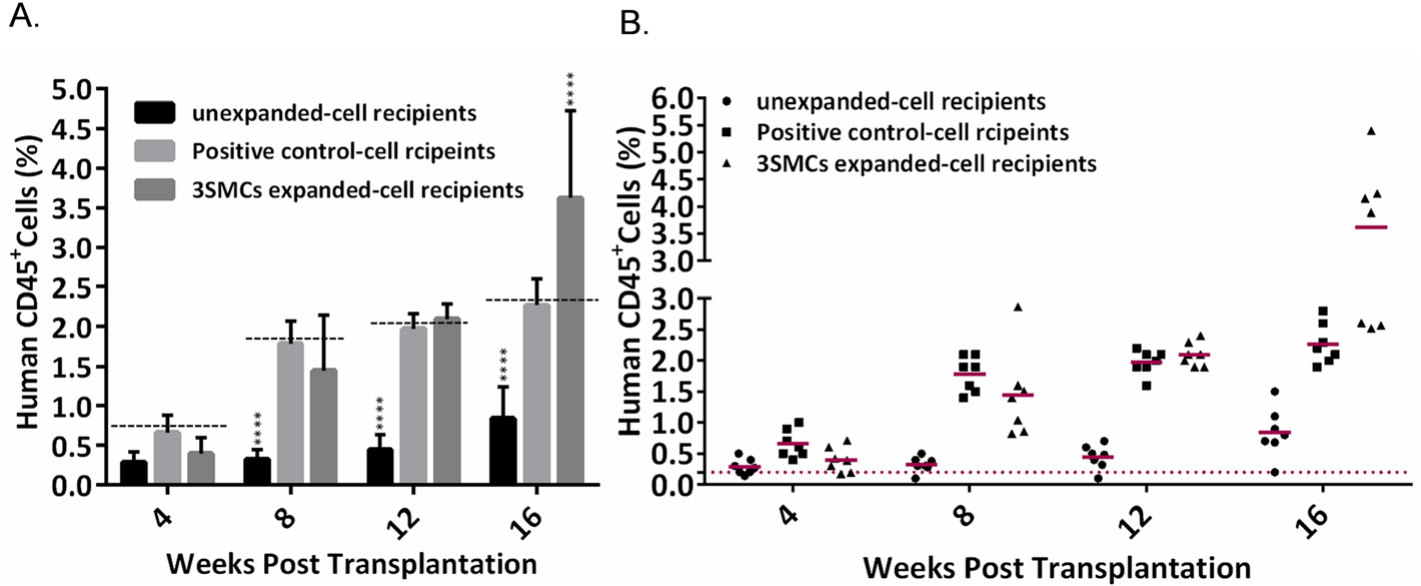
The level of expanded hUCB-CD34+ cells engraftment in the peripheral blood of NMRI mice. **a** The percentage of human CD45 cells in the peripheral blood of newborn mice. Each bar indicated mean ± SD for at least 6 independent samples. ****P ≤ 0.0001. **b** Each shape indicates the percentage of human CD45 expression in the peripheral blood of one newborn mouse. Mice with ≥ 0·2% human cells were considered chimeric

In order to evaluate the in vivo functional capability of the expanded CD34^+^ cells, we used in utero transplantation model [23]. We transplanted 30–50 × 10^3^ freshly isolated hUCB-CD34^+^ cells or the cells harvested from the cultures with the same number of input hUCB-CD34^+^ cells in the presence or absence of SM cocktail into NMRI mouse embryos, E11.5-E13.5. Two weeks after birth, born mice were treated with human hematopoietic growth factors SCF (4 ng/g), IL-3 (4 ng/g), and G-CSF (50 ng/g) for 1 week. As shown in Fig. 3, by treatment with the human hematopoietic factor, the hCD45^+^ chimerism was distinctly increased compared with initial values, 4 and 8 weeks post transplantation. Sixteen weeks after transplantation, the average human cell engraftment in the peripheral blood of the mice transplanted with freshly isolated hUCB CD34^+^ cells was about 1%, while the percentage of CD45^+^ cells in 3SMs and positive control transplanted mice was 9 times and 3.4 times (3.6 ± 1 and 3.2 ± 0.3) respectively, compared to the unexpanded cell recipients (Fig. 3). In the other words, the ex vivo expansion of hUCB CD34^+^ cells with SM cocktail resulted in 1.5 fold increase in human cell engraftment compared to the positive control.

### Ability of the optimal SM cocktail to modulate the cell signaling pathways

**Fig. 4:**
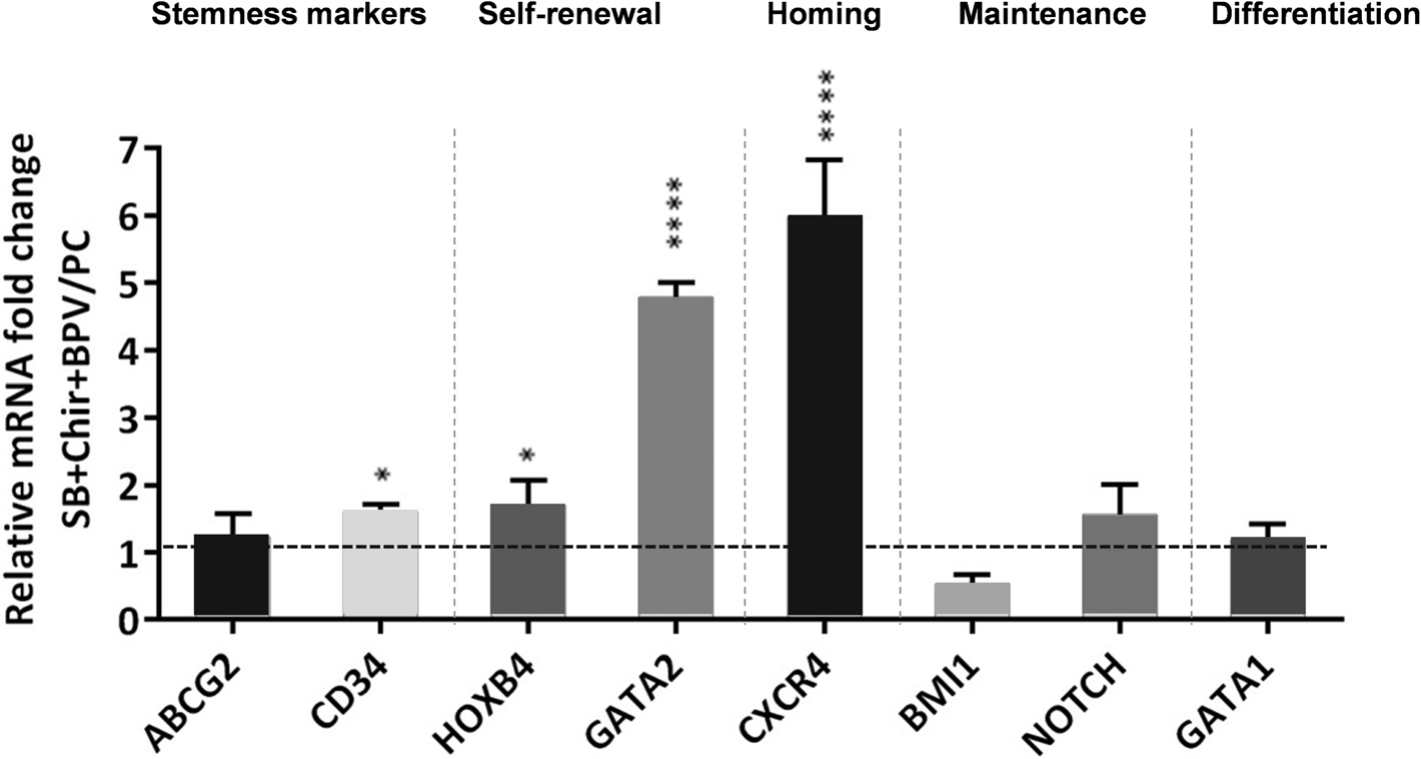
Treatment by SB, Chir, and Bpv modifies the gene expression of UCB-CD34^+^ cells. Bars represent the mean fold-changes of gene expression in the 3 SM-expanded cells relative to the positive control group detected by quantitative real-time PCR (*n* = 3), *P ≤ 0.05, **P ≤ 0.01, ***P ≤ 0.001 vs. positive control

Subsequently, RT-qPCR was performed in order to determine the expression of typical genes involved in HSC stemness. The result shows that the relative expression of the two major genes involved in the proliferation and self-renewal of HSCs, including *HOXB4* and *GATA2* as well as the HSC-specific marker, CD34, have significantly increased in the presence of 3SM cocktail after normalization to the level of the PC group. Furthermore, the expression of the *CXCR4* gene involved in the migration and transplantation of HSCs has increased dramatically in the presence of 3SM cocktail. The expression of other genes associated with self-renewal, such as *ABCG2*, *Notch*, and Bmi1, does not show a significant difference between the groups (Fig. 4).

## Discussion

In recent years, small molecules have been widely used in the field of stem cell research. So far, there have been numerous indications for the successful use of small molecules to inhibit apoptotic and differentiation processes during hematopoietic stem cell reproduction [8]. It seems that a combination of two or more small molecules may produce a better result. For example, the effect of chir and insulin [10], chir, and rapamycin [24] along with SCF, TPO, and Flt3L has been reported to enhance the proliferation of mouse hematopoietic stem cells. The proliferation of human hematopoietic stem cells has also been studied in the presence of various combinations of 5Aza, TSA, VPA, and NAM [25–27]. Notably, based on the cell status, synergistic/antagonistic interactions may have been created between the small molecules. As a result, the simultaneous use of small molecule compounds can produce unpredictable results compared to their individual use. To our knowledge, this is the first study in which the expansion of CD34^+^ cells is targeted through the simultaneous modulation of proliferation, differentiation, and apoptosis signaling pathway.

In this study, a cocktail of seven small molecules was selected to target the TGFβ, ERK, Wnt, Akt, Hedgehog, and P53 signaling pathways as well as the cell epigenome. Then, their best combination to induce efficient HSC expansion was screened through the eliminative approach. To the successful expansion of UCB-CD34^+^ cells, SCF, TPO, and Flt3L which greatly affect the HSC signaling pathways were also added to the culture medium. Our experiments conducted us to this notion that the addition of SB, Chir, and Bpv to the HSC conventional HSC culture medium increases the efficiency of ex vivo expansion of CD34^+^ cells with 50-fold enhancement in the number of CD34^+^ 38^−^ cells. The small molecule cocktail can also augment the colony formation ability of expanded cells (Fig. 2). All these changes were associated with the upregulation of HOXB4, GATA2, and CD34 gene. Moreover, here, higher engraftment potential and higher percentage of human CD45 cells in infused mice confirm the in vivo potential of the expanded cells in the presence of small-molecule cocktail.

**Fig. 5:**
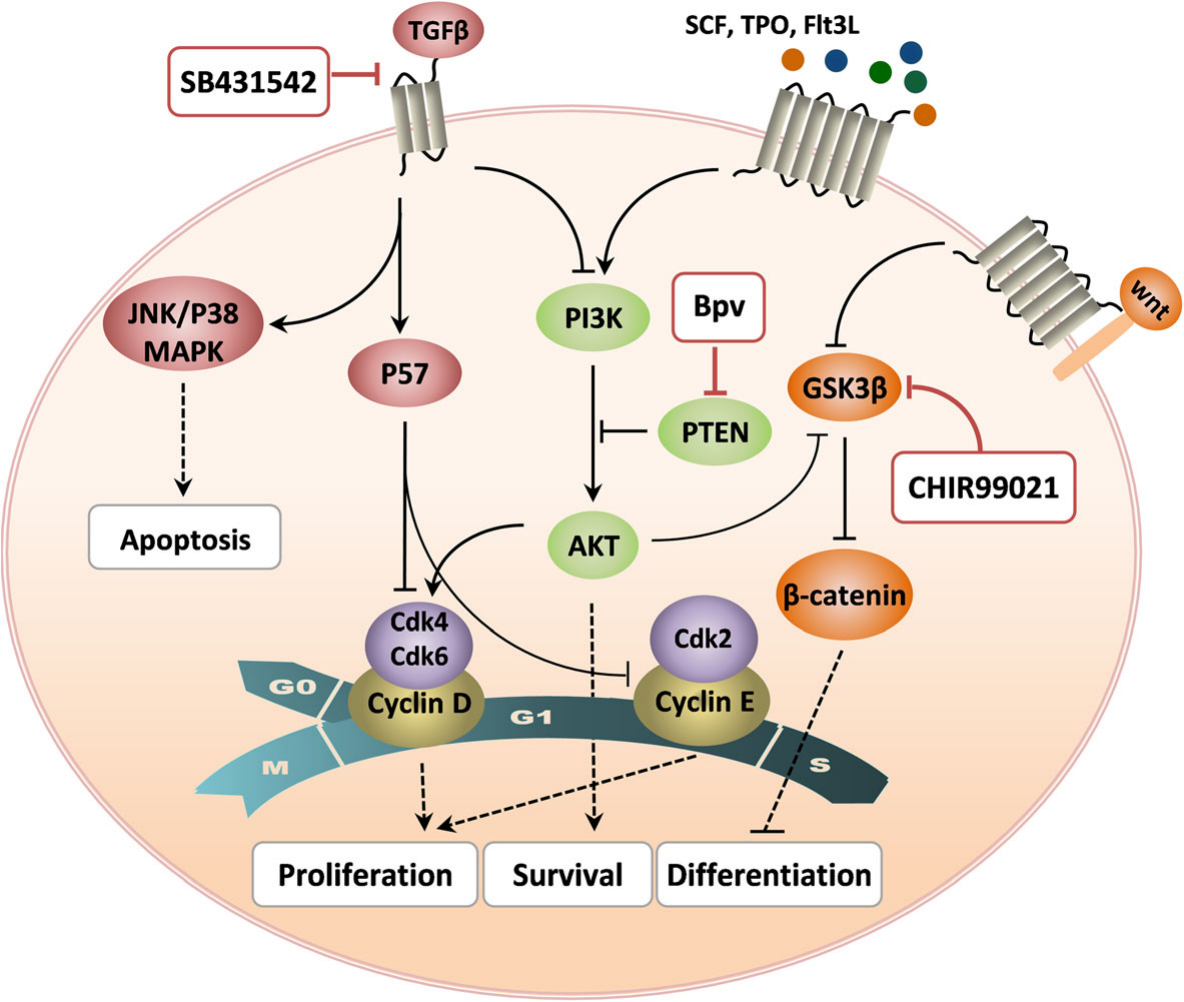
The molecular mechanisms which through them SB, Chir, and Bpv modulate proliferation, differentiation, and survival of hematopoietic stem cells

According to our findings, the best result is obtained by simultaneous controlling of PTEN/Akt, Wnt/β-catenin, and TGFβ signaling pathways in such a way that Bpv leads to exiting the cells from the quiescence and proliferation through inhibiting PTEN and enhancing the Akt pathway. On the other hand, Chir indirectly inhibits the differentiation process through GSK3 inhibition and β-Catenin activation. All of these events occur while TGFβ, the most important apoptotic pathway, is inhibited by SB (Fig. 5).

PI3K-AKT pathway is one of the most important pathways affecting a wide range of stem cell cellular signaling molecules [28]. In particular, many apoptotic proteins such as Bim and Bcl-2 can be inactivated by the pathway. AKT, also, inhibits certain cell cycle inhibitors such as P21 and P27 and activates Cyclin D, which in turn leads to exit from G0 and entry into the cell cycle [29]. Furthermore, Akt facilitates the migration of HSCs and their binding to the bone marrow stromal cells through induction of integrin expression [30, 31]. PTEN is a tumor suppressor protein that inhibits the PI3K-AKT pathway. Actually, inhibition of PTEN leads to increased survival, proliferation, self-renewal, and incomplete differentiation potential of embryonic stem cells [32] and also in vitro proliferation of HSCs [10].

Wnt pathway not only plays a critical role in the development of embryonic stem cells [33], but also in the proliferation and differentiation of adult stem cells including HSCs [34, 35]. The major effects of Wnt are applied through β-catenin which can increase the self-renewal and proliferation of HSCs, even independently of the Wnt pathway [36, 37]. According to previous studies, the accumulation of the β-catenin, following GSK3 inactivation, facilitates the maintenance of the pluripotency state of embryonic and adult stem cells [38, 39].

TGFβ is one of the major negative regulators of HSC proliferation [40]. The pathway, specifically, inhibits cell cycle progression through the induction of P57 expression, which in turn leads to CyclinD-Cdk4/6 and CyclinE-Cdk2 inactivation. P38MAPK is also a downstream molecule of the TGFβ pathway which its inhibition results in decreased in vitro apoptosis and aging of HSCs [40]. JNK is another downstream target of TGFβ which activates some apoptotic factors such as Bcl2 and Bad. Therefore, inhibition of the TGFβ pathway not only leads to P57, P38MAPK, and JNK inhibition which is associated with cell cycle promotion, but also inhibits the apoptotic pathways [41–43].

Altogether, a cocktail of SB431542, Chir99021, and Bpv, which respectively inhibits the TGFβ differentiation pathway and activates the Wnt and Akt pathways, can be used to improve the conventional protocol of HSC expansion.

## Supplementary information

**Additional file 1: Figure S1.** Schematic illustration of procedure to find the best combination of small molecules to expand UCB-HSCs. **Table S1.** Initial concentration of small molecules based on previous studies and their proper concentration based on MTS assay. **Table S2.** List of primer sequences used in the present study.

## Data Availability

All data generated or analyzed during this study are included in this published article and in supplementary figures.

## Abbreviations

UCB: Umbilical cord blood
HSCs: Hematopoietic stem cells
SMs: Small molecules
SB: SB431542
Pur: Purmorphamin
PD: PD0325901
Chit: Chir9901
Bpv: Bisperoxovanadium
Pμ: Pifithrin-μ
NAM: Nicotinamide
CFU: Colony-forming units
BFU-E: Burst-forming unit-erythroid
CFU-GM: CFU granulocyte-macrophage
CFU-GEMM: CFU granulocyte-erythrocyte-macrophage-megakaryocyte
