# Supplementary material for: Combination of SB431542, Chir9901, and Bpv as a novel supplement in the culture of umbilical cord blood hematopoietic stem cells
Source: Stem Cell Res Ther. 2020 Nov 9;11:474. doi: 10.1186/s13287-020-01945-8 (PMC7650159; doi:10.1186/s13287-020-01945-8)
Supplement: Supplementary file 1 — Additional file 1: Figure S1. Schematic illustration of procedure to find the best combination of small molecules to expand UCB-HSCs. Table S1. Initial concentration of small molecules based on previous studies and their proper concentration based on MTS assay. Table S2. List of primer sequences used in the present study. [file 13287_2020_1945_MOESM1_ESM.docx]

**Supplementary information**

**Figure S1.** Schematic illustration of procedure to find the best combination of small molecules to expand UCB-HSCs.

| **Set**  **Concentration** | **Initial**  **Concentration** | **References** | **Small molecules** |
| --- | --- | --- | --- |
| 10 μM | 10 μM | 10.1007/s12015-013-9473-0 | **SB431542 (SB)** |
| 0.25 μM | 1 μM | 10.1007/s12015-013-9473-0 | **PD0325901 (PD)** |
| 4 μM | 4 μM | 10.1371/journal.pone.005649 | **Purmorphamin (19)** |
| 0.3 μM | 3 μM | 10.1007/s12015-013-9473-0 | **Chir99021 (Chir)** |
| 2.5 mM | 2.5 mM | 1016/j.exphem.2011.12.005 | **Nicotinamide (NAM)** |
| 5 μM | 5 μM | 10.1111/j.1474-9726.2011.00704.x. | **Bpv** |
| 2.5 μM | 10 μM | 10.1186/1476-4598-10-115 | **Pifithrin-µ (Pμ)** |

**Table S1.** Initial concentration of small molecules based on previous studies and their proper concentration based on MTS assay.

| Gene Name | Primer sequence (5’ 3’) | TM (°C) | Size (bp) |
| --- | --- | --- | --- |
| *GAPDH* | F: CTC ATT TCC TGG TAT GAC AAC GA  R: CTT CCT CTT GTG CTC TTG CT | 121 | 59 |
| *RUNX1* | F: CCA CTC CAC TGC CTT TAA CC  R: GGT ATT GGT AGG ACT GAT CGT | 199 | 60 |
| *FOG1* | F: CCG CAG TGA TCA ACA AAG AC  R: CTC TTT GGG CTT CTC GTC TG | 155 | 60 |
| *BMI1* | F: GTG TGC TTT GTG GAG GG  R: AGT AGT GGT CTG GTC TTG TG | 148 | 60 |
| *HOXB4* | F: AGA CAG AAA GAG AAA TAG GAG G  R: CGG CAG AGG AAA CAA GAC | 126 | 60 |
| *LMO2* | F: AAC TGG GCC GGA AGC TCT  R: CTT GAA ACA TTC CAG GTG ATA CA | 143 | 65 |
| *TAL1* | F: CTC GGC AGC GGG TTC TTT G  R: CGT CTT GCA GGA GGT CAT CTG | 94 | 61 |
| *GATA1* | F: GGG ATC ACA CTG AGC TTG C  R: ACC CCT GAT TCT GGT GTG G | 202 | 60 |
| *GATA2* | F: AAG GCT CGT TCC TGT TCA GA  R:GGC ATT GCA CAG GTA GTG G | 102 | 60 |
| *PU1* | F: CAC AGC GAG TTC GAG AGC TT  R: GAT GGG TAC TGG AGG CAC AT | 194 | 61 |
| *OCT4* | F: GTT CTT CAT TCA CTA AGG AAG G  R: CAA GAG CAT CAT TGA ACT TCA C | 148 | 60 |
| *JUN* | F: GGT AGC AGA TAA GTG TTG AG  R: GGG CAG TTA GAG AGA AGG | 185 | 60 |
| *CXCR4* | F: AAC TTC AGT TTG TTG GCT GC  R: CAT TTC CTC GGT GTA GTT ATC TG | 142 | 60 |

**Table S2.** List of primer sequences used in the present study.
